# **“**Like an umbrella, protecting me from the rain until I get to my destination”: Evaluating the implementation of a tailored primary care model for urban marginalized populations

**DOI:** 10.1186/s12875-024-02563-6

**Published:** 2024-09-28

**Authors:** Soha Khorsand, Carol Geller, Alison Eyre, Hounaida Abi Haidar, Haifeng Chen, Corina Lacombe, Monisha Kabir, Andrew Mclellan

**Affiliations:** 1 Faculty of Medicine, University of Ottawa, Ottawa, ON Canada; 2 Department of Family Medicine, Faculty of Medicine, University of Ottawa, 201-600 Peter Morand Crescent, Ottawa, ON K1G 5Z3 Canada; 3 Centretown Community Health Centre, Ottawa, ON Canada; 4 Department of Psychology, University of Ottawa, Ottawa, ON, Canada; 5 Bruyère Research Institute, Ottawa, ON Canada; 6 Lawrence S. Bloomberg Faculty of Nursing, University of Toronto, Toronto, ON Canada

**Keywords:** Primary Health Care, Community Health Centers, Social Marginalization, Qualitative Research

## Abstract

**Background:**

Improving health equity and access to the highest possible standard of health care is a key issue of social accountability. Centretown Community Health Centre in Ottawa, Canada has iteratively developed a program to target and serve marginalized and complex populations since 1999. The program implementation was evaluated using a validated implementation framework.

**Methods:**

Quantitative and qualitative data were collected through a health records extraction (*n* = 570), a client complexity assessment tool (*n* = 74), semi-structured interviews with clients and key stakeholders (*n* = 41), and a structured client satisfaction survey (*n* = 30). Data were analyzed using descriptive statistics and inductive thematic analysis.

**Results:**

Five hundred and seventy unique clients were seen between November 1–30, 2021. A third of clients (34%) did not have a provincial health card for access to universal health care services, and most (68%) were homeless or a resident of rooming houses. Most clients who reported their income (92%) were at or below Canada’s official poverty line.

The total mean complexity score for clients seen over a one-month period (*n* = 74) was 16.68 (SD 6.75) where a total score of at least 13 of 33 is perceived to be a threshold for client biopsychosocial complexity. Clients gained the majority of their total score from the Social support assessment component of the tool.

Clients (*n* = 31) and key informants (*n* = 10) highlighted the importance of building relationships with this population, providing wrap-around care, and providing low-barrier care as major strength to the Urban Health program (UH). Key areas for improvement included the need to: i) increase staff diversity, ii) expand program hours and availability, and iii) improve access to harm reduction services. Clients appeared to be highly satisfied with the program, rating the program an average total score of 18.50 out of 20.

**Conclusions:**

The program appears to serve marginalized and complex clients and seems well-received by the community. Our findings have relevance for other health care organizations seeking to better serve marginalized and medically and socially complex individuals and families in their communities.

**Supplementary Information:**

The online version contains supplementary material available at 10.1186/s12875-024-02563-6.

## Background

### Primary care

Primary care is the entry point to health services for most health problems. A high quality primary care system can contribute to a reduction in avoidable hospitalization, better health outcomes, a decrease in mortality rates related to socioeconomic disparities, and higher life expectancy [1]. Quality of care is the extent to which health care services improve the probability of desired health outcomes being achieved for individuals and populations [2]. Further, quality of care can be measured and improved over time through the delivery of evidence-based care that acknowledges and addresses service-user (patient/client) experiences [2]. High quality primary care has been linked to increased access to services, improved delivery of care, and prevention and early management of health conditions [1]. In order to provide high quality care in primary care settings, health care providers must address health problems, provide client navigation through the health care systems, ensure a continuous relationship with clients, prevent and identify health conditions early, and establish links between community resources and clients and their families [3].

### Marginalized and complex populations

Historically, persistently, or systematically marginalized populations are those who experience barriers to engaging in mainstream social, economic, cultural, or political life [4, 5]. Potential reasons for their marginalization may include aspects of their identity (i.e., gender, culture, language, race, sexual orientation) and socioeconomic status [4]. Further, this may include individuals who experience hardship due to homelessness (e.g., no fixed address), substance use, and prior involvement with the correctional system. Finally, groups such as Indigenous peoples, people with mental illnesses, LGBTQIA + and racial minorities (including immigrants/refugees) may also experience marginalization [6]. Urban marginalized populations often share common social determinants of health related to being unable to access social systems and/or experiences of poverty [6]. To serve the needs of marginalized populations, it is essential for health care providers to deliver both the indicated care for the client’s condition and proactively designed individualized care targeted towards specific sources of complexity and social determinants of health [7].

Client “complexity” can be defined as barriers to standard health care provision experienced by clients due to the severity of their psychosocial and medical conditions, lack of social safety, and lack of coordination of care or negative care system relationships [8]. Client complexity is linked to social determinants of health, wherein social and environmental elements contribute to an individual’s or community’s health risks [8]. Thus, social determinants of health may contribute to both marginalization and complexity [6, 8]. Complexity affects clinical presentations, the choice and organization of care, and clinical outcomes. Identifying and addressing the client's complexity can indicate the kind and level of team structure, care management, and social services involvement needed to provide quality primary care.

### Providing care to marginalized and complex populations

Challenges to accessing primary care services have been documented for urban marginalized and complex individuals [9–11]. In Canada, economic, geographic, cultural, and language barriers may limit access to health care [9, 10]. Further, many clinics and specialists require proof of public health insurance for billing which hinders access and treatment options for those who face barriers to attaining identification documentation. A client’s lack of an address, personal identification documents, access to transportation, telephone, and social support to negotiate and navigate the system may contribute to barriers in accessing appropriate and timely care [6].

Low access to preventative medical care for this population has been associated with more complex presentations, higher morbidity and mortality, higher rates of emergency department visits, and inpatient admissions [12]. Additionally, stigmatization and discrimination within the health care system can contribute to reluctance to accessing services, contributing to a preventable worsening of health outcomes [6, 13].

Equitable access to primary care requires health care providers and organizations to consider social and health determinants, the needs of the proposed clients, and resources available to provide services to those that need it [14]. Barriers preventing access include the method in which care is offered as well as the ability of individuals to perceive, seek, reach, pay for, and engage with care [15]. The need for an integrated team approach is well-documented, and improves expertise, resources, shared responsibilities, and client-centered case management [6, 13, 16, 17].

Therefore, the purpose of this study was to evaluate the implementation of a program developed to meet the needs of urban marginalized populations in one service area in Ottawa, Canada. We evaluated the implementation of the program using client health records data, a provider-rated client complexity assessment, semi-structured interviews, and a client satisfaction survey. The questions guiding this evaluation were as follows:

1. What is the quality of the implementation of the program in terms of its services and client perceptions?
2. Does the implemented program align with its intended aim of engaging and caring for marginalized and complex populations?

## Methods

### Setting/context

The Centretown Community Health Centre (CCHC) is a community health centre in Ottawa, Canada with a catchment area that includes the city’s largest family shelter for refugee claimants, a young men’s shelter, a women’s shelter, as well as numerous rooming houses. This made for an ideal setting to evaluate the implementation of a primary care program for marginalized populations.

### Process of program design

An Urban Health (UH) program was created to provide high quality, comprehensive, and interprofessional primary care services and tailored to target the needs of the urban and marginalized individuals in the community. Specifically, the program aimed to address the changing population demographics in the community health centre’s surrounding area, including large populations of people with lower income and immigrant status [18].

The UH program has evolved over the last 24 years. The program emerged organically through relationships between case managers and primary care providers working in the same space, and often serving similar clients. The need for designing a tailored program to provide holistic and equitable care for marginalized communities was recognized. The planning, development and operationalization of the program occurred through a series of interprofessional staff meetings (including with family physicians, nurse practitioners, case workers).

The objective of the program was to reduce barriers to the healthcare system for the most marginalized populations in Ottawa. Previous research conducted by the Centretown Community Health Centre helped to identify barriers in accessing care for marginalized and new immigrant and refugee populations [19]. The program now includes: a) flexible, no appointment primary care clinics; b) appointment-based services including evening access for clients and families when an appointment model serves them best; c) social supports services encompassing harm reduction, case management/coordination, social/healthcare system navigation, shower access, coaching, and counseling; d) on-site access to psychiatric services; and e) outreach social work and nursing services, on the streets, in rooming houses, and in shelters (specifically Ottawa women’s, family, and youth shelters).

### Framework and reporting

To evaluate the implementation of the UH program, we aimed to also evaluate the health service quality and program fidelity. This was done with the goal of ensuring the program provides high quality primary care services and reaches its intended target population. The Proctor et al. [20] program implementation framework was utilized to guide the evaluation. The framework examines three distinct but interrelated outcomes: implementation, service, and client. These outcomes evaluate whether the program resembles what was intended when it was launched and if the program has reached its goals, indicating successful implementation. Service outcomes measure the quality and characteristics of the service delivery. Client outcomes measure the program users’ experience.

Implementation outcomes are positioned as preceding both service and client outcomes [21]. Theoretically, the more positive the implementation outcomes (i.e., if the program was implemented successfully), the more positive the service and client outcomes.

A subset of outcomes from the Proctor et al. framework were used to evaluate the UH program. They are defined below in Table 1.

**Table 1:** Outcomes used to evaluate the Urban Health program implementation from Proctor et al. [20]

Type of outcome	Term	Definition
Implementation	Appropriateness	The perceived fit, relevance, or compatibility of the evidence-based intervention for a particular setting, as well as the fit of the intervention to address a particular issue or problem
	Acceptability	The perception among implementation stakeholders that a given practice is agreeable, palatable, or satisfactory
	Penetration	The degree to which a practice has been integrated into a service setting and its subsystems
	Fidelity	The degree to which an intervention was implemented as it was intended by the program developers, typically by comparing the original evidence-based intervention and the disseminated/implemented intervention in terms of adherence to the program protocol, amount of program delivered, and quality of program delivery
Service	Person-centredness	The degree to which an individual client’s specific health needs and desired health outcomes are considered in health care decisions and quality measurements. It encompasses qualities of compassion, empathy, and responsiveness to the needs, values, and expressed preferences of the individual client
	Effectiveness	The ability of an intervention to have a meaningful effect on clients in normal clinical conditions
	Equity	The degree to which a program provides a fair opportunity for clients to attain their highest level of health, regardless of their income, social status, race, gender, and education. [22]
Client	Satisfaction	A subjective measure of whether a client’s expectations about a health encounter were met

### Evaluation methods

The study involved using an embedded mixed methods design [23] to assess the implementation of the UH program. The evaluation of the UH program implementation involved four separate components and data sources, as detailed in Table 2.

**Table 2:** Description of evaluation methods, data sources, and data types

Evaluation method	Data source	Data type	Description
1. Client health record extraction	Community health centre’s electronic medical records	Quantitative	One author (HC) extracted client information from electronic medical records for a 1-year period (July 1, 2020 – June 30, 2021). The dataset was able to be filtered for specific demographic and medical categories (e.g., could search codes for specific chronic conditions). This author reviewed the data extracted for randomly selected individual clients for accuracy (5% of total client charts). In addition, all the authors examined the full dataset for irregularities during analysis Extracted items included access to a provincial health card for access to universal health care services, housing situation, income level, chronic conditions, key barriers experienced (e.g., immigration issues, language barriers,), and Urban Health program services accessed
2. Client complexity assessment	The Health Connection Clinic Complexity Assessment (AMPS) Tool [24] (derived from the Minnesota Complexity Assessment Method [8] which was in turn adapted from the validated INTERMED tool [25–28])	Quantitative	The 11-item tool is organized through the acronym AMPS, referring to four main areas of complexity: Attachment; Medical; Psychiatric; and Social. The AMPS is a clinician-rated measure that is scored on a Likert scale ranging from 0 (descriptor) to 3 (descriptor). Health care providers are instructed to rate each item based on their patient encounter. The Attachment score evaluates whether clients are rostered to a general practitioner. The Medical score evaluates the severity of a client's medical symptoms and degree of regular management required. The Psychiatric score evaluates severity and management of mental health and addictions symptoms. The Social score evaluates client’s access to housing, food, predictable income, social support, and subjective cooperativity with the health and social care system. Item scores are summed to create a total score. A total score of at least 13 of 33 is generally perceived to be a threshold for client biopsychosocial complexity A consecutive sample of all clients seen at the UH program clinic for the period of one month (November 1–30, 2021) was utilized. The assessment was conducted following each client appointment by the primary care provider. Only one assessment was done for each client no matter the number of clinic visits during the month
3. Semi-structured interviews	Interviews	Qualitative	**Clients** Program clients were interviewed in English or French or by using phone translation services in other languages for approximately 60 min. Interviews focused on participants' perceptions of the program's appropriateness, acceptability, effectiveness, fidelity, and person-centredness Participants were recruited via convenience sampling at the program clinic. Informed consent was obtained from each participant following the research team providing client participants with study information and the rationale for conducting the study. Participants were only included if they had at least three interactions with the program within the last five years. One individual (SK) conducted in-person interviews using an interview guide (Additional File 1) developed by the research team with simultaneous note taking. All interviews were audio-recorded and transcribed verbatim **Key informants** Key informant interviews were conducted with UH program staff and personnel from partnering agencies (i.e., drop-ins, soup kitchens, and homeless shelters) at their workplaces to gather qualitative data about perceptions and experiences with the program. Interviews took place for approximately 60 min Key informants were recruited via purposive sampling. Written informed consent was obtained from each key informant following the research team providing key informants with study information and the rationale for conducting the study. One individual (SK) conducted the in-person interviews using an interview guide (Additional File 2) developed by the research team with simultaneous note taking. All interviews were audio-recorded and transcribed verbatim
4. Client satisfaction	Client Satisfaction Questionnaire^©^ (CSQ-8) [29, 30]	Quantitative	The 8-item CSQ-8 was used to measure client satisfaction. The tool is meant to be unidimensional, yielding a homogeneous estimate of general satisfaction with services [30]. The five questions (items #1, 2, 4, 7–8) most applicable to the aims of our study were embedded into the end of client semi-structured interviews

All components of this study were approved by the Ottawa Health Science Network (Protocol #: 2090011-01H) research ethics board on October 21, 2019.

### Data analysis

Descriptive statistics were calculated for clients’ demographic characteristics (e.g., age, gender, immigration status, insurance, housing situation, length of time involved with the program, etc.) and client satisfaction with the UH program. Client demographics were used to identify which populations accessed the UH program.

Qualitative data were explored via inductive thematic analysis [31]. Thematic analysis is a method of systematically identifying, organizing, and offering insight into patterns of meaning (themes) that allows researchers to make sense of collective or shared meaning and experiences [31], without being limited to a particular theoretical or epistemological approach [32]. The interview transcripts were subjected to initial coding, defined as a process of breaking down, examining, comparing, conceptualizing, and categorizing data [31]. Themes and subthemes were then identified through constant comparison [31]. Two researchers (SK and HA) trained in qualitative research methodology conducted coding independently, with regular meetings to discuss coding discrepancies and generate themes based on similarities or differences within and across the dataset. NVivo 12 [33] was used to code interview transcripts and identify the main themes and subthemes. The Strengthening the Reporting of Observational Studies in Epidemiology (STROBE) Statement [34] and Consolidated Criteria for Reporting Qualitative Research (COREQ) [35] was used to report this study’s findings.

## Results

### Evaluation

#### Client health records extraction

Between July 1, 2020—June 30, 2021, 570 unique clients were seen, with 1859 total client visits (mean 3.26, SD 4.10) to the UH Program clinic. A third of clients (34%) did not have a provincial health card for access to universal health care services, and most (68%) were homeless or a resident of rooming houses. Of the 219 individuals who reported their income (not reported: *n* = 351), 92% were at or below Canada’s official poverty line ($0–14,999/year: 81%, $15,000–19,000/year: 11%). The most common chronic illnesses included substance use disorder (30%), psychiatric disorder(s) (29%), hypertension (18%), diabetes (10%), latent tuberculosis (7%), chronic hepatitis B or C (6%), and HIV (2%). Additional concurrent issues ranged from immigration issues (27%), issues with refugee claims (26%), problems with the healthcare system (20%), and language barriers (17%). The UH program services used by clients included medical (84%), social assistance (i.e., access to showers, clothing, general health system navigation, etc.; 84%), and mental health (26%) services.

#### Client complexity assessment

The AMPS score was calculated for all clients seen in the UH program between November 1–30, 2021 (*n* = 74). The total mean complexity score for clients seen during this time was 16.68 (SD 6.75) out of 33. On average, male clients (*n* = 35; mean 18.66, SD 5.85) had higher mean total scores than female clients (*n* = 37; mean 14.65, SD 7.11). The highest recorded AMPS score was in the 40–49 age group (*n* = 13; mean 21.31, SD 5.12). Table 3 presents the AMPS complexity score broken down by age and sex.

**Table 3:** Client AMPS complexity score by a) age range and b) sex between November 1–30, 2021

a)																													
Age, years	**AMPS complexity score (maximum possible points)**																												
	**Total (33)**					**A: Attachment (3)**						**M: Medical (6)**						**P: Psychiatric-mental-health-addictions (12)**							**S: Social support (12)**				
	Mean	SD	Median			Mean		SD		Median		Mean		SD		Median		Mean			SD		Median		Mean		SD		Median
Total	16.68	6.75	17.00			2.95		0.37		3.00		2.45		1.26		2.00		3.68			3.63		3.00		6.09		3.16		6.00
0–19	14.67	9.95	10.50			2.83		0.41		3.00		1.33		1.21		1.50		3.33			5.32		0.00		6.00		4.34		5.50
20–29	14.18	6.88	13.00			3.00		0.00		3.00		1.55		1.04		2.00		3.36			3.44		2.00		4.91		3.59		3.00
30–39	16.25	6.34	16.00			3.00		0.00		3.00		2.17		0.39		2.00		3.92			4.29		3.00		5.50		2.54		6.00
40–49	21.31	5.12	22.00			3.00		0.00		3.00		2.54		1.20		2.00		6.31			3.25		8.00		7.70		2.89		9.00
50–59	17.15	5.89	18.00			3.00		0.00		3.00		3.23		1.42		3.00		2.54			2.96		1.00		6.15		2.76		5.00
60–69	14.57	6.93	14.00			3.00		0.00		3.00		2.93		1.07		2.50		2.50			2.35		2.50		5.79		3.42		6.00
≥ 70	17.00	5.52	16.00			2.40		1.34		3.00		2.80		1.64		3.00		3.60			4.16		3.00		6.60		2.88		8.00
b)																													
Sex		**AMPS complexity score (maximum possible points)**																											
		**Total (33)**					**A: Attachment (3)**						**M: Medical (6)**						**P: Psychiatric-mental-health-addictions (12)**					**S: Social support (12)**					
		Mean		SD	Median		Mean		SD		Median		Mean		SD		Median		Mean	SD		Median		Mean		SD		Median	
Total (*N* = 74)		16.68		6.75	17.00		2.95		0.37		3.00		2.45		1.26		2.00		3.68	3.63		3.00		6.09		3.16		6.00	
Male (*n* = 35)		18.66		5.85	19.00		2.91		0.51		3.00		2.46		1.42		2.00		5.17	3.63		5.00		6.57		2.73		6.00	
Female (*n* = 37)		14.65		7.11	12.00		2.97		0.16		3.00		2.46		1.14		2.00		2.14	3.04		0.00		5.57		3.54		5.00	
Other or blank (*n* = 2)		19.50		3.54	19.50		3.00		0.00		3.00		2.00		0.00		2.00		6.00	2.83		6.00		7.50		2.12		7.50	

The mean attachment score for all clients was 2.95 out of 3.00 (SD 0.37), reflecting the inclusion criteria of the UH program, which specifically targets individuals and families with no primary care provider. Medical complexity had a mean score of 2.45 out of 6.00 (SD 1.26), suggesting that clients had medical symptoms which were typically well-controlled and stabilized. Psychiatric (mental health and addictions) complexity had a mean score of 3.68 out of 12.00 (SD 3.63), suggesting clients had mild to moderate mental health and addictions symptoms and management. The Social section had a mean of 6.09 out of 12.00 (SD 3.16), suggesting a moderate to high degree of poverty in the population, as well as low access to social supports, and hesitant attitude towards care providers. For the most part, clients gained the majority of their total score from the Social component of the tool, which may be reflective of the social marginalization of clients seen through the UH program.

#### Semi-structured interviews

Thirty-one clients and ten key informants (program staff and staff of partnering agencies) consented to participate in the semi-structured interviews. One client withdrew their consent to participate at the midpoint of the interview. Responses provided by this interviewee were included up to the point of withdrawal. Client demographics are presented in Table 4. Key informants from four different organizations participated in interviews.

**Table 4:** Demographics for clients who participated in interviews (*n* = 31)

Characteristic (row %)	Mean (SD)	*N* (%)
Age (years; *n* = 31)	45.97 (12.7)	
Sex (*n* = 31)		
Male		23 (74.2)
Female		8 (25.8)
Family status (*n* = 31)		
Single		18 (58.1)
Living with partner		2 (6.5)
Family		11 (35.5)
Immigration status (*n* = 31)		
Immigrant		9 (29.0)
Refugee		9 (29.0)
Non-immigrant (Canadian)		11 (35.5)
Indigenous		2 (6.5)
Country of origin (*n* = 31)		
Canada		13 (41.9)
Burundi		4 (12.9)
Rwanda		3 (9.7)
Kenya		1 (3.2)
Djibouti		1 (3.2)
Somalia		1 (3.2)
Congo		1 (3.2)
Senegal		1 (3.2)
Nigeria		1 (3.2)
Morocco		1 (3.2)
Iraq		1 (3.2)
Bangladesh		1 (3.2)
Soviet Union^a^		1 (3.2)
Poland		1 (3.2)
Current living/housing situation (*n* = 30)		
No fixed address		2 (6.7)
Shelter		1 (3.3)
Subsidized housing		10 (33.3)
Rooming/boarding house		14 (46.7)
Renting		3 (10.0)
Financial situation in terms of food (*n* = 31) Not difficult making ends meet		10 (32.3)
Difficult making ends meet—some of the time		10 (32.3)
Difficult making ends meet—most of the time		5 (16.1)
Difficult making ends meet—all of the time		6 (19.4)
Financial situation in terms of shelter (*n* = 31)		
Not difficult making ends meet		17 (54.8)
Difficult making ends meet—some of the time		5 (16.1)
Difficult making ends meet—most of the time		6 (19.4)
Difficult making ends meet—all of the time		3 (9.7)
Financial situation in terms of predictable income (*n* = 31)		
Not difficult making ends meet		5 (16.1)
Difficult making ends meet—some of the time		11 (35.5)
Difficult making ends meet—most of the time		6 (19.4)
Difficult making ends meet—all of the time		9 (29.0)
Financial barriers to getting health care needed (*n* = 30)		
Yes, all of the time		3 (10.0)
Yes, most of the time		9 (30.0)
No, most of the time		4 (13.3)
Never		14 (46.7)

^a^Country designation when participant emigrated

During interviews, both clients and key informants highlighted the importance of building relationships with this population, providing wrap-around care, and providing low-barrier care as major strengths of the UH program. These findings support the idea that the program is appropriate, effective, person-centred, and acceptable to clients, which ultimately reflects the fidelity of the program’s iterative development. Themes and representative quotes are provided in Table 5.

**Table 5:** Themes identified from semi-structured interviews with clients (*n* = 31) and key informants (*n* = 10)

**Theme**		**Definition**	**Representative quotes**
Building relationships	Client perspective	Client reflections on feeling at ease with care providers	4B: “I like the atmosphere: the way that people treat me. People are empathetic, kind, helpful, and non-judgmental.” 9B: “Centretown is safe, no judgment zone. Feels like home.” 13A: “Centretown has been like an umbrella, protecting me from the rain until I get to my destination.”
	Key informant perspective	Statements by staff of partnering agencies highlighting the importance of building relationships with the population	8C: “building relationships and trust is key in this population, for clients that know us well, it works really well because they have trusting relationships. clients do not feel judged when they come in” 1C: “… building rapport with the clients, being non-confrontational. Providing dignity and respect in a non-judgmental manner. Having [an outreach nurse] come in, she puts the clients at ease, she immerses herself in with the clients, becomes part of the group. Provide as much dignity and respect.”
The importance of wrap-around care (medical, psychosocial, advocacy)	Client perspective	Instances where clients highlighted the availability of multiple services as main attractants to the program	13A: “Good services here, give you everything you need.” 4A: “Very professional here, they send reminder calls, everyone treats me well. very organized, and everyone coordinates with each other, I don't feel lost here”
	Key informant perspective	Instances where key informants highlighted the availability of multiple services as reasons why they refer their clients to the program Instances where program staff discuss how their roles include providing wrap-around care	10C: “[The] health center has a little bit of something for everyone: counseling, [identification document supports], substance abuse, harm reduction. something for everyone, especially the homeless and transient population. good feedback about the staff, word of mouth is that if you go down the street they will help you.” 7C: “… often our clients are less able to advocate for their needs, we can take time and advocate for their needs. For example with [Ontario Disability Support Program] worker, housing registry, helping access services that might have barriers that are preventing them from accessing at the time.”
Access to low-barrier care	Client perspective	This theme encompasses the removal of financial, physical, and social barriers in order to allow access to high quality care based on clients’ perspectives	9A: “Other places will not accept the refugee papers. I have to pay upfront and pay additional fees [making it] difficult to access. [Refugee health coverage] is limited in what they cover, and I am unemployed, so that makes everything harder.” 1B: “other clinics have too many clients, they are very busy, too many people”
	Key informant perspective	This theme encompasses the removal of financial, physical, and social barriers in order to allow access to high quality care based on key informants’ perspectives	8C: “For people who have difficulty accessing traditional services- we attempt to bridge the gap in access by bringing healthcare to them and connecting them to community health centre.” 9C: “… people who aren’t able to independently access walk-in that others who have insurance can access. We refer them to the community health centre”
Room for improvement	Client perspective	Client reflections on areas that need improvement within UO	14B: “programs are hard to access because only [a] certain number of clients can be seen and if I don’t get in on time, I will not be seen. One more day/week would be good.”
	Key informant perspective	Ideas from program staff and partners regarding how to better serve the community	6C: “… having more multicultural staff, staff who can speak different languages (i.e. Arabic). I would love to see staff who speak more than just French and English (i.e., Arabic, Somali) … people working at the program are white, clients ask about why there are no black nurses. Sometimes the clients are scared. People are spending 16–24 months in the shelter, but only see white nurses. Clients get discouraged about the ‘multiculturality’ of Canada, when they only see white staff. Many clients don’t say everything to the nurse because of that. Sometimes they come into my office and tell me information because I am black.” 8C: “I have noticed we don’t offer much in terms of addictions medicine (OAT, safer supply). We don’t have a safe injection site (lots of overdoses in rooming houses, people just outside the building needing monitoring for overdoses). Biggest glaring gap is support for people with addictions.*”* 1C: “Our clients have a hard time accessing the Urban health clinic. Clients have a hard time getting through reception: ‘i.e., do you have a family doctor? If yes, can’t come in’. If we send five clients there, four will be sent back, have to take 2–3 extra steps.”

#### Client satisfaction

An adapted version of the CSQ-8^©^ was embedded into the end of the client semi-structured interviews (*n* = 30; *n* = 1 withdrew midway through the interview). Clients were asked to score each item on a Likert scale of 1 to 4, with a total possible maximum score of 20 across the 5 items. Overall, clients within our sample appeared to be highly satisfied with the program, rating the program an average total score of 18.50 out of 20. Score breakdown is shown in Table 6.

**Table 6:** Client satisfaction ratings (*N* = 30) of the Urban Health (UH) program

Variable (total score)	Mean score (SD)
1. Quality of service (4)	3.80 (0.48)
2. Type of service (4)	3.53 (0.78)
3. Recommend to a friend (4)	3.73 (0.58)
4. Overall satisfaction (4)	3.67 (0.71)
5. Come back (4)	3.77 (0.62)
***Total (20)***	***18.50 (2.43)***

### Summary of evaluation and associated implementation outcomes

As previously mentioned, four evaluation methods were used to evaluate the success of the implementation of the UH program: client health record extraction (*n* = 1859), client complexity assessments (*n* = 74), semi-structured interviews (*n* = 31), and a client satisfaction survey (*n* = 30). Each tool served as a measure of one or more of the Proctor et al. implementation outcomes, as outlined below in Table 7.

**Table 7:** Overview of evaluation methods and associated outcomes from the Proctor et al. [20] Implementation Framework

**Evaluation method**		**Proctor et al. ** [20]** outcomes**	
1. Client health record extraction		● Fidelity	The program evolved with the intention to target the most socially vulnerable clients in the catchment area. The health record extraction demonstrated that the majority of the clients seen in one year were experiencing homelessness or did not have access to health insurance The program was successful in adhering to the original program design (fidelity)
2. Client complexity assessment		● Appropriateness ● Fidelity	The program evolved with the intention to target the most socially vulnerable clients in the catchment area The client complexity assessment demonstrates a high degree of client complexity in terms of the majority of clients having low access to primary care and lacking social support services (appropriateness) The program was successful in adhering to the original program design (fidelity)
3. Semi-structured interviews	Theme 1: Building relationships	● Acceptability ● Person-centredness	Clients and partnering program staff discussed the importance and success of client-staff relationship building at the program and reflected positively on their overall experiences with the program Key stakeholders in the program found it to be agreeable (acceptability) Clients also discussed feeling empathy, compassion, and responsiveness from their care providers (person-centredness)
	Theme 2: The importance of wrap-around care	● Appropriateness ● Penetration ● Effectiveness ● Fidelity	Clients and staff of partnering agencies reflected on the particular utility of having multiple services in one place for the population served (appropriateness) Key informants reflected on the program as being one that they knew of to send their clients to if their clients needed help (penetration) Clients of the program reflect positively on being able to receive holistic care, and feeling well taken care of (effectiveness) The program evolved with the intention to provide holistic care, and this theme supports the idea that it was successful in doing so (fidelity)
	Theme 3: Access to low barrier care	● Fidelity ● Equity	The program evolved to provide clients with access to care without having to face barriers present in the traditional health care system Clients reflect positively on being able to access care at UH because the program accepts immigrant and refugee clients without provincial health coverage and provides walk-in services (fidelity) Key informants also discuss a gap in the traditional health care system and discuss the way that UH is used to fill that gap successfully (equity)
4. Client satisfaction rating		● Satisfaction	Program clients are found to be highly satisfied with the program (satisfaction)

## Discussion

Overall, based on the evaluation methods used to assess the implementation of the UH program, clients and key informants found the program to demonstrate fidelity, appropriateness, person-centredness, equity, acceptability, effectiveness, and penetration. This evaluation indicates a successful implementation and evolution of the program, including achieving goals such as removing barriers to accessing health care for marginalized, complex individuals and families, and high client satisfaction with the program’s services. Key areas for improvement that were identified as a result of this work include the need to: i) increase diversity in staff to better represent and serve the population served, ii) expand program hours and availability to allow for better accessibility to services, and iii) improve access to harm reduction services within the program to better serve the population that uses substances.

Our study found that the client population served through Centretown Community Health Centre’s UH program is highly complex. We used the Health Connection Clinic Complexity Assessment Tool to quantify the complexity of clients by examining their attachment to a provider, medical conditions and management, psychiatric conditions and management, and social situation. This tool was developed and initially used at a community health centre serving clients living in Vancouver’s Downtown Eastside neighbourhood, which contains Canada’s lowest income postal code, and is at the epicentre of the national opioid crisis [36, 37]. Presumably, this population is one of the most complex in Canada with respect to biopsychosocial complexity. In the paper reporting on the development of the Health Connection Clinic Complexity Assessment Tool, program staff assigned an AMPS score to every client that was seen in their clinic over the course of 1 year, and found a mean complexity score of 14.38 [24]. In contrast, in applying the AMPS score to every client seen through the UH program over the course of one month, we found a total mean complexity score that was higher, with even higher mean scores when evaluating just male clients or those in the 40–49-year-old age group. This demonstrates that the complexity of the clients served by the UH program is comparable to one of the most complex populations in Canada.

Based on the results from our review of UH program clients’ electronic medical records, the majority of the program users are at or below the poverty line and a third did not have access to a provincial health card (i.e., likely immigrants/newcomers or individuals with no fixed address). According to Employment and Social Development Canada’s Poverty Reduction Strategy report [38], Canada’s official poverty line ranges from $16,436 to $20,639 of annual income for single individuals, depending on one’s geographical location. Given that 21.5% of single adults in Canada were living in poverty in 2020 and that those with immigrant or refugee status are more likely to be living in poverty [39], there is a clear need for services similar to the UH program across Canada to address the needs of these marginalized groups. The development and implementation of services targeting marginalized individuals, including those with low income or immigrant or refugee status is crucial given prior evidence of barriers to accessing health care [40–43] and economic impacts related to the COVID-19 pandemic disproportionately affecting these populations [44, 45].

In interviews conducted as part of this study, clients and key informants highlighted the importance of fostering a judgment-free space, building relationships and trust, and having access to low-barrier care involving multiple services. These findings are echoed by other community programs offering similar services for marginalized groups [46, 47] with an aim to reduce health care inequities. Overcoming the barriers faced by complex and marginalized communities require an integrated approach of understanding and addressing the social determinants of health affecting these groups in care delivery [4, 48] and collaborative, coordinated care services [6]. Accordingly, based on client and key informant perceptions of the UH program, societal factors such as health care coverage, income, access to care services, and housing are acknowledged, and clients’ individual needs are advocated for through the person-centred approach and wrap-around model of care offered by the program. Despite some areas of improvement being noted by participants, these results demonstrate the success of the program’s implementation, and indicate approaches that similar programs could take to ensure the needs of their clients are met.

The UH program’s objective is to reach urban marginalized population, and the complexity scores of its clients and low-income status supports the notion that this intended aim has been reached. Our findings are consistent with the fact that the service area of the UH program includes a neighbourhood with a high-prevalence of low-income constituents and a high index of socioeconomic disadvantage when compared to other neighbourhoods in Ottawa, Canada [49]. Given that most major urban centres have populations facing poverty, substance use, unstable housing, food insecurity, and new immigrant/refugees, we anticipate that our findings will be beneficial for other service providers seeking to develop and evaluate the implementation of programs serving urban marginalized populations.

### Strengths and limitations

A key strength of this study is that it provides support for the development and implementation of a program targeting the needs of urban and marginalized individuals in the community, including those with lower income or immigrant status. Similar programs may be developed and implemented using our description of how the UH program was designed and our evaluation of its implementation.

The following limitations of this study must be considered. Interviews were conducted at one point in time, and therefore may not have captured the potentially evolving perspectives and needs of participants. Moreover, it is possible that individuals who were more enthusiastic about sharing their experiences, whether positive or negative, may have been more likely to participate in the interviews and client satisfaction assessment. This may have potentially introduced some bias into our findings. Client satisfaction ratings were collected at the end of semi-structured interviews. Therefore, our client satisfaction results are specific to the small sample of individuals who took part in interviews and may not be generalizable across all UH program clients. We attempted to ameliorate these limitations by recruiting a diverse range of individuals for participation in interviews.

Additionally, interviews for this project were completed during the summer of 2021, when many COVID-19 restrictions were still in place. At that time, many social services which are integral to the experience of UH program clients (i.e., social support groups, community cooking lessons, etc.) were put on hold, meaning that clients were not able to comment on these services’ impacts on their experience. It is also possible that screening for COVID symptoms at the health centre door may have excluded groups of clients with general poorer health from entering the health centre, and therefore having an opportunity to be recruited to the study. This may have contributed to more healthy clients being represented in our interview findings.

Another limitation may have been the use of the Health Connection Clinic Complexity Assessment Tool for the assessment of client complexity. While the AMPS score is theoretically sophisticated and robust [24, 25], in recent years it has been criticized due to the subjectivity of questionnaire methodology [50]. Instead, a new software tool has been created by the developers of the Health Connection Clinic Complexity Assessment Tool. This new software tool models the complexity of clients by complementing questionnaire data with analysis of electronic health record data from the community health centre, as well data from other service settings used by their clients throughout the city including other community health centers, detox centers, and emergency departments [51]. Unfortunately, the program does not currently have access to electronic health records at other services throughout the city, and therefore using a software similar to this was not possible for this project. We did however aim to mitigate the limitations of questionnaire subjectivity by holding a provider training session on the use of this tool prior to its use. We additionally used data from the program’s electronic health record to complement the AMPS results.

Lastly, in our experience, mental illness, the impact of substances on behavior and health care seeking approaches, and the impact of past experiences with the health care system, influence interactions between service providers and marginalized populations. Medical mistrust and potential impacts of sharing information, especially in marginalized populations, may have a strong negative influence on health system interactions [52–55]. Our tools did not capture and quantify these aspects.

Despite these limitations, this study adds to the literature on the development, implementation and evaluation of programs to address equitable access to primary care services for urban marginalized and complex populations. More research is needed to assess how marginalization and complexity affects primary care outcomes.

## Conclusions

This study evaluated the implementation of a holistic, interprofessional program at a community health centre to address unmet needs of urban marginalized individuals who have historically encountered barriers to accessing quality health care. Based on our evaluation, we have found that the program is serving its intended population of individuals who do not have public system coverage for health care, are at or below the national poverty line, suffer from a myriad of chronic illnesses, and have high complexity as determined by attachment, medical, psychiatric, and social parameters. Client and key informant perceptions of the program were exceedingly positive though some areas of improvement were noted to enhance care delivery. Our findings have relevance for other health care organizations and care providers seeking to create and evaluate the implementation of their own programs to better serve urban marginalized individuals in their communities and reduce health care inequities.

## Supplementary Information

Supplementary Material 1

Supplementary Material 2

Supplementary Material 3

Supplementary Material 4

## Data Availability

The de-identified dataset used and analyzed during the current study are available from the corresponding author on reasonable request. To preserve the anonymity of the interviewees, the transcribed interviews are not available for sharing.
