# Supplementary material for: “Like an umbrella, protecting me from the rain until I get to my destination”: Evaluating the implementation of a tailored primary care model for urban marginalized populations
Source: BMC Prim Care. 2024 Sep 28;25:347. doi: 10.1186/s12875-024-02563-6 (PMC11437737; doi:10.1186/s12875-024-02563-6)
Supplement: Supplementary file 1 — Supplementary Material 1 [file 12875_2024_2563_MOESM1_ESM.pdf]

Additional File 1: Interview Guide with Clients, Phase I

Participant ID Number: C _

Date of Survey (dd/mm/yyyy):

Thank you for agreeing to participate in this survey. The purpose of this interview is to learn about your experiences at the Centretown Community Health Centre. Your answers can help us improve our programs and services. We are interested in your honest opinions, whether they are positive or negative. Comments and suggestions are also welcome. Please answer the questions to the best of your abilities.

First, I want to ask you a couple of personal questions:

1. Where are you from? ____
    a. If outside of Canada:
    i. When did you move here? __________________________
    ii. How long have you lived here? ____ (days/weeks/moths/years)
    iii. Are you a refugee, immigrant, etc.?
    a. Yes
    b. No

2. What is your current living/housing situation?
    a. Shelter
    b. Subsidized housing
    c. Rooming/boarding house
    d. Street
    e. Not disclosed

3. How would you characterize your financial situation in terms of food, shelter, predictable income or employment No difficult making ends meet

- ❑ Difficult making ends meet- some of the time
- ❑ Difficult making ends meet- most of the time
- ❑ Difficult making ends meet- all of the time
- ❑ Other: ____

4. Is there any financial barriers to you getting the health care you need?

- ❑ Yes all of the time
- ❑ Yes most of the time
- ❑ No most of the time
- ❑ Never

5. How would you assess your general health status compared to that of others of your own age?

- ❑ Much better
- ❑ Slightly better
- ❑ Neither better nor worse
- ❑ Slightly worse
- ❑ Much worse

Now, I will ask you some questions about the Centretown Community Health Centre:

6. How did you first hear/learn about the Centretown Community Health Centre? (*Check all that apply.)*
   - ❑ Outreach workers
   - ❑ Shelter staff
   - ❑ From close others who use CCHC
   - ❑ In the community
   - ❑ Other programs or centres
   - ❑ Brochures/flyers
   - ❑ Other: ____

7. What are your reasons for visiting the Centretown Community Health Centre? *(Check all that apply and specify.)*
   - ❑ Medical/physical health: ______________________________
   - ❑ Mental health: ___________________________________
   - ❑ Support: ____
   - ❑ Assistance: ____

8. What made CCHC a place you would return to?

____
____
____
____
____

9. When something was needed for you (like a referral) did the program get it in a timely manner?

____
____
____
____
____

10. Did the program hours allow you to access servicers? If not why? Any suggestions?

____
____
____
____
____

11. If anything what does the center/program do to discourage you coming back?
____
____
____
____
____

12. Where have you accessed health care in Ottawa/Ontario besides at Centretown?
____
____
____
____
____

13. Have you had any reasons/challenges for accessing health care elsewhere?
____
____
____
____
____

14. Where have you accessed health care in Ottawa besides at Centretown?

____

15. Have you had any reasons/challenges for not accessing health care elsewhere?

____

_

16. [In the past], which services have you used at the Centretown Community Health Centre? *(Circle all that apply and list associated services.)*
❑ Medical/physical health: ____________________________________
❑ Psychiatry: ____
❑ Support: ____
❑ Practical Assistance: _______________________________________
❑ Harm reduction:___________________________________

17. In the past year, have the doctors or nurses asked if you would like to receive any cancer screening or immunizations or blood work?
    i. Yes
    ii. No

18. If yes, what types of cancer screening? *(Check all that apply.)*
    ❑ Colonoscopy

❑ Pap test
❑ Mammogram/breast cancer screening

Finally, I'm going to ask you a few questions about your level of satisfaction with the services and programs at CCHC:

[Selected questions from the Client Satisfaction Questionnaire (CSQ-8): https://csqscales.com/product/csq-8/]

That concludes our interview. Thank you very much for your time!

[Proceed to Quality Check on following page.]

Quality Check:

Immediately following the interview, please complete the following report.

Rate the quality of the interview on a scale from 1 (not at all) to 4 (very).

	**Not at all**	**Slightly**	**Moderately**	**Very**
The participant was focused/undistracted	1	2	3	4
The participant understood the questions	1	2	3	4
The participant effectively communicated their responses	1	2	3	4
The participant was cooperative	1	2	3	4
The participant was confident in their answers	1	2	3	4

What were your overall feelings/impressions of this interview?

____

Additional comments:
