# Supplementary material for: “Like an umbrella, protecting me from the rain until I get to my destination”: Evaluating the implementation of a tailored primary care model for urban marginalized populations
Source: BMC Prim Care. 2024 Sep 28;25:347. doi: 10.1186/s12875-024-02563-6 (PMC11437737; doi:10.1186/s12875-024-02563-6)
Supplement: Supplementary file 2 — Supplementary Material 2 [file 12875_2024_2563_MOESM2_ESM.pdf]

Additional File 2: Interview Guide with Key Informants, Phase I

Participant ID Number: I_ _

Date: _______________

The purpose of this interview is to discuss the Urban Outreach (UO) programs recruitment processes and outreach targets. I will ask you a series of questions about your experiences with the UO program and its client characteristics. Please answer each question to the best of your abilities. You have the right to refuse any question(s) and/or stop this interview at any time. At the end of the interview, you will have the opportunity to give me any additional feedback. You can withdraw at any time during this interview, and refuse to answer any questions you do not feel comfortable doing so.

1. First, I want you to think about what does a typical outreach day look like? Who do you think the UO program serves?
    a. Which shelter(s) do you visit?
        i. Which populations do you interact with?
    b. What do your tasks involve?
2. Can you describe the clients who you refer or you believe typically utilize the services at UO program?
    a. What are their backgrounds (i.e., are newcomers to Canada)?
    b. What is their housing situation?
    c. Which services are they utilizing?
3. Do you notice any gaps in in the UO program?
    a. Are there certain groups/populations that are difficult to reach?
    b. Are there certain groups/populations that rarely engage or avoid UO programming?
        i. Can you describe them?
4. What do you think attracts certain clients to use the UO program?
    a. Which factors might discourage clients from using the Urban Outreach?
5. How do you encourage the UO program's intended targets to participate and utilize the programs and services at CCHC?
    a. Which techniques have been successful in the past?
        i. What are some successful techniques that you currently employ?
    b. What would you change about, or implement, at UO program to encourage future participation?
    c. What would you do to improve the UO program?

That concludes our interview. Thank you very much for your time!

(Turn off tape, and ask if they would like to give any feedback or comments off-record.)
