# Supplementary material for: “Like an umbrella, protecting me from the rain until I get to my destination”: Evaluating the implementation of a tailored primary care model for urban marginalized populations
Source: BMC Prim Care. 2024 Sep 28;25:347. doi: 10.1186/s12875-024-02563-6 (PMC11437737; doi:10.1186/s12875-024-02563-6)
Supplement: Supplementary file 3 — Supplementary Material 3 [file 12875_2024_2563_MOESM3_ESM.docx]

# Consolidated criteria for Reporting Qualitative Research (COREQ) Checklist (Tong et al., 2007)

| **Item** | **Guide questions/description** | **Response and page number referenced** |
| --- | --- | --- |
| *Domain 1: Research team and reflexivity* | |  |
| 1. Interviewer/ facilitator | Which author/s conducted the interview or focus group? | Page 12:  “Two researchers (SK and HA) trained in qualitative research methodology conducted coding independently, with regular meetings to discuss coding discrepancies and generate themes based on similarities or differences within and across the dataset.” |
| 1. Credentials | What were the researcher’s credentials? e.g. PhD, MD | Researcher credentials are provided in the author list. |
| 1. Occupation | What was their occupation at the time of the study? | The researchers conducting data collection and analysis were both employed for the study. |
| 1. Gender | Was the researcher male or female? | This detail was not included in the manuscript as the interviewer’s gender did not have a bearing on the content of interviews. |
| 1. Experience and training | What experience or training did the researcher have? | Page 12:  “Two researchers (SK and HA) trained in qualitative research methodology conducted coding independently, with regular meetings to discuss coding discrepancies and generate themes based on similarities or differences within and across the dataset.” |
| 1. Relationship established | Was a relationship established prior to study commencement? | N/A |
| 1. Participant knowledge of the interviewer | What did the participants know about the researcher? e.g. personal goals, reasons for doing the research | Table 2, “3. Semi-structured interviews” row:  “Informed consent was obtained from each participant following the research team providing client participants with study information and the rationale for conducting the study.”  “Written informed consent was obtained from each key informant following the research team providing key informants with study information and the rationale for conducting the study.” |
| 1. Interviewer characteristics | What characteristics were reported about the interviewer/facilitator? e.g. Bias, assumptions, reasons and interests in the research topic | Table 2, “3. Semi-structured interviews” row:  “Informed consent was obtained from each participant following the research team providing client participants with study information and the rationale for conducting the study.”  “Written informed consent was obtained from each key informant following the research team providing key informants with study information and the rationale for conducting the study.” |
| *Domain 2: Study design* | |  |
| 1. Methodological orientation and Theory | What methodological orientation was stated to underpin the study? e.g. grounded theory, discourse analysis, ethnography, phenomenology, content analysis | Page 12:  “Qualitative data were explored via inductive thematic analysis [24]. Thematic analysis is a method of systematically identifying, organizing, and offering insight into patterns of meaning (themes) that allows researchers to make sense of collective or shared meaning and experiences [24], without being limited to a particular theoretical or epistemological approach [25].” |
| 1. Sampling | How were participants selected? e.g. purposive, convenience, consecutive, snowball | Table 2, “3. Semi-structured interviews” row:  “Participants were recruited via convenience sampling at the program clinic.”  “Key informants were recruited via purposive sampling.” |
| 1. Method of approach | How were participants approached? e.g. face-to-face, telephone, mail, email | Table 2, “3. Semi-structured interviews” row:  “One individual (SK) conducted in-person interviews using an interview guide (Additional File 1) developed by the research team with simultaneous note taking.”  “One individual (SK) conducted in-person interviews using an interview guide (Additional File 1) developed by the research team with simultaneous note taking.” |
| 1. Sample size | How many participants were in the study? | Page 16:  “Thirty-one clients and ten key informants (program staff and staff of partnering agencies) consented to participate in the semi-structured interviews. One client withdrew their consent to participate at the midpoint of the interview. Responses provided by this interviewee were included up to the point of withdrawal. Client demographics are presented in Table 4. Key informants from four different organizations participated in interviews.” |
| 1. Non-participation | How many people refused to participate or dropped out? Reasons? | Page 16:  “Thirty-one clients and ten key informants (program staff and staff of partnering agencies) consented to participate in the semi-structured interviews. One client withdrew their consent to participate at the midpoint of the interview. Responses provided by this interviewee were included up to the point of withdrawal. Client demographics are presented in Table 4. Key informants from four different organizations participated in interviews.” |
| 1. Setting of data collection | Where was the data collected? e.g. home, clinic, workplace | Table 2, “3. Semi-structured interviews” row:  “Participants were recruited via convenience sampling at the program clinic.”  “Key informant interviews were conducted with UH program staff and personnel from partnering agencies (i.e., drop-ins, soup kitchens, and homeless shelters) at their workplaces to gather qualitative data about perceptions and experiences with the program.” |
| 1. Presence of non-participants | Was anyone else present besides the participants and researchers? | Table 2, “3. Semi-structured interviews” row:  “One individual (SK) conducted in-person interviews using an interview guide (Additional File 1) with simultaneous note taking.”  “One individual (SK) conducted the in-person interviews using an interview guide (Additional File 2) with simultaneous note taking.” |
| 1. Description of sample | What are the important characteristics of the sample? e.g. demographic data, date | Page 16:  “Client demographics are presented in Table 4. Key informants from four different organizations participated in interviews.” |
| 1. Interview guide | Were questions, prompts, guides provided by the authors? Was it pilot tested? | Table 2, “3. Semi-structured interviews” row:  “One individual (SK) conducted in-person interviews using an interview guide (Additional File 1) developed by the research team with simultaneous note taking.”  “One individual (SK) conducted in-person interviews using an interview guide (Additional File 1) developed by the research team with simultaneous note taking.” |
| 1. Repeat interviews | Were repeat interviews carried out? If yes, how many? | N/A |
| 1. Audio/visual recording | Did the research use audio or visual recording to collect the data? | Table 2, “3. Semi-structured interviews” row:  “All interviews were audio-recorded and transcribed verbatim.” |
| 1. Field notes | Were field notes made during and/or after the interview or focus group? | While field notes were not created, the individual conducting in-person interviews took notes throughout interviews. |
| 1. Duration | What was the duration of the interviews or focus group? | Table 2, “3. Semi-structured interviews” row:  “Program clients were interviewed in English or French or by using phone translation services in other languages for approximately 60 minutes.”  “Interviews took place for approximately 60 minutes.” |
| 1. Data saturation | Was data saturation discussed? | N/A |
| 1. Transcripts returned | Were transcripts returned to participants for comment and/or correction? | Transcripts were not returned to participants. Instead, the trustworthiness of the data collection and analysis procedures was ensured by conducting data collection and analysis in an iterative way with two analysts. |
| *Domain 3: Analysis and findings* | |  |
| 1. Number of data coders | How many data coders coded the data? | Page 12:  “Two researchers (SK and HA) trained in qualitative research methodology conducted coding independently, with regular meetings to discuss coding discrepancies and generate themes based on similarities or differences within and across the dataset.” |
| 1. Description of the coding tree | Did authors provide a description of the coding tree? | Page 12:  “Qualitative data were explored via inductive thematic analysis [24]. Thematic analysis is a method of systematically identifying, organizing, and offering insight into patterns of meaning (themes) that allows researchers to make sense of collective or shared meaning and experiences [24], without being limited to a particular theoretical or epistemological approach [25]. The interview transcripts were subjected to initial coding, defined as a process of breaking down, examining, comparing, conceptualizing, and categorizing data [24]. Themes and subthemes were then identified through constant comparison [24].” |
| 1. Derivation of themes | Were themes identified in advance or derived from the data? | Page 12:  “Qualitative data were explored via inductive thematic analysis [24].” |
| 1. Software | What software, if applicable, was used to manage the data? | Page 12:  “NVivo 12 [26] was used to code interview transcripts and identify the main themes and subthemes.” |
| 1. Participant checking | Did participants provide feedback on the findings? | N/A |
| 1. Quotations presented | Were participant quotations presented to illustrate the themes / findings? Was each  quotation identified? e.g. participant number | Participant quotations are presented in Table 5 along with participant numbers and descriptions of each theme. |
| 1. Data and findings consistent | Was there consistency between the data presented and the findings? | The findings were derived from the data using an inductive approach (see page 12), and therefore are consistent with the data presented. |
| 1. Clarity of major themes | Were major themes clearly presented in the findings? | Major themes are clearly presented in Table 5, along with representative quotations. |
| 1. Clarity of minor themes | Is there a description of diverse cases or discussion of minor themes? | Themes that were less prevalent within and across the dataset (e.g., “Room for improvement” across the client dataset) are clearly presented in Table 5 where representative participant quotations are provided for each theme. |

**From:** Tong A, Sainsbury P, Craig J. Consolidated criteria for reporting qualitative research (COREQ): a 32-item checklist for interviews and focus groups. International Journal for Quality in Health Care. 2007. Volume 19, Number 6: pp. 349 – 357
